# Another “Lock-Jaw” Case

**Published:** 1861-06

**Authors:** 


### Selections.

Another "Lock-jaw" Case.—*Messrs. Editors:*—In your Journal for Nov. 17th, you allude to a ridiculous mistake in diagnosis, and mention a case of dislocated jaw that, by a homœopath, was mistaken and treated for *lock-jaw*, with the jaw *locked* open? That case brings to my mind another. When I was a six-month student of medicine, a neighbor, while vomiting, dislocated his lower jaw. He consulted an *eclectic*, who, after an hour's fruitless attempt at reduction, told him he was engaged to go out with a company on a pleasure excursion, and he would give the case further attention on his return. Not being willing to wait, the patient consulted a homœopath. He, too, made a fruitless attempt at reduction. He then gave the patient some of his globules to be taken, and a liquid with which to bathe the face, and left, promising to renew the effort at reduction on the morrow, providing it did not replace itself in the meantime! Half an hour later, I saw the patient. He presented a ludicrous aspect, and, indeed, seemed like one poorly resigned to his fate. He couldn't *jaw* his previous medical attendants, for the reason his jaw wouldn't work. I offered my services, (students are not amenable to the code), and reduced the dislocation without difficulty. When the homœopath renewed his visit, and learned the condition of things, he took glory to himself by saying that no man living could have reduced that dislocation, *if he had not given medicine to "relax the cords!"* The aspirant for surgical glory left abruptly when the patient informed him that none of the medicine left had been used! —*Medical and Surgical Reporter.* O. C. G.

